# Supplementary figures and images for: Effects of BRD4 inhibitor JQ1 on the expression profile of super-enhancer related lncRNAs and mRNAs in cervical cancer HeLa cells
Source: PeerJ. 2024 Feb 23;12:e17035. doi: 10.7717/peerj.17035 (PMC10896078; doi:10.7717/peerj.17035)

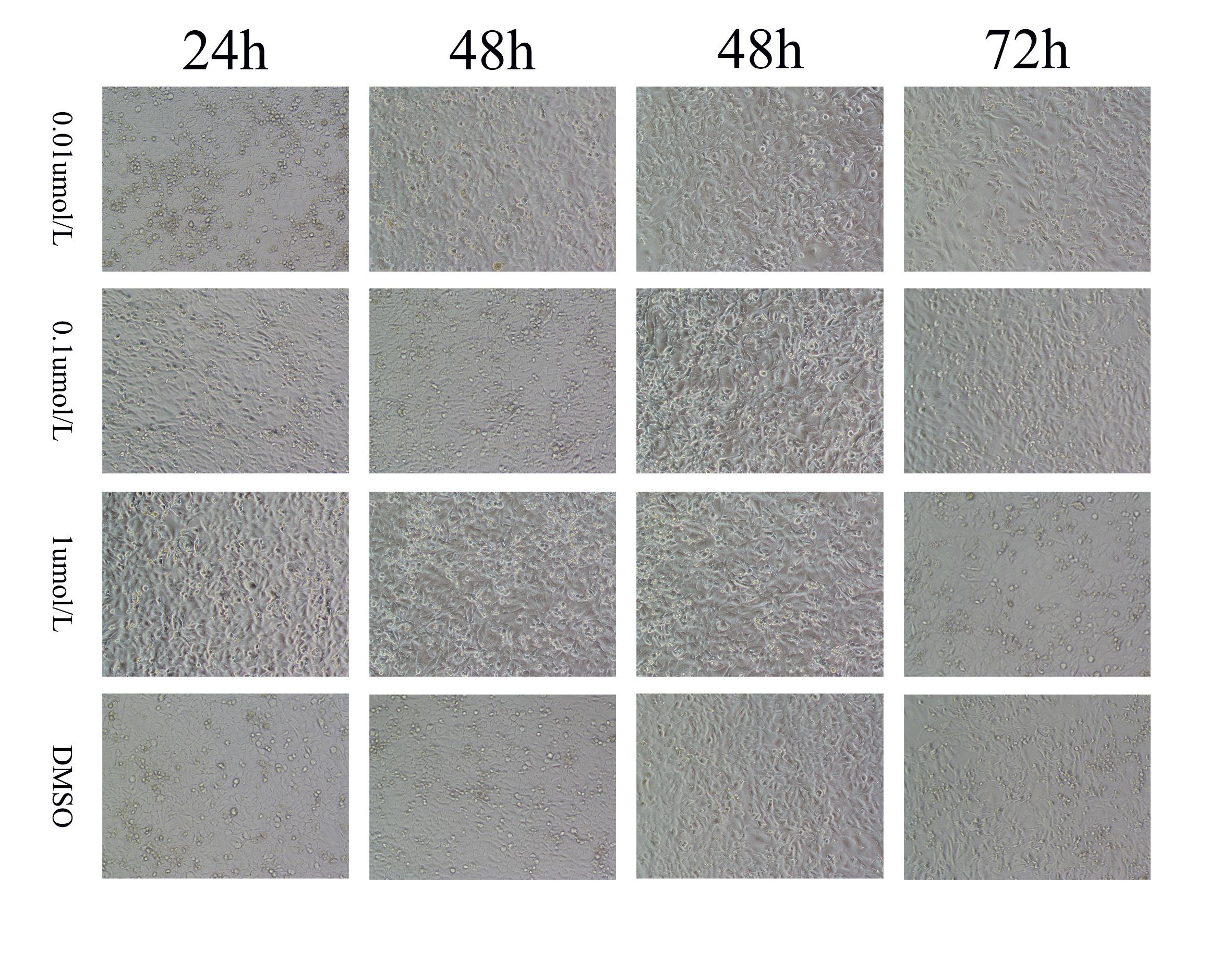

Supplement: Supplemental Information 4 [file peerj-12-17035-s004.png]
