# Supplementary material for: Effects of BRD4 inhibitor JQ1 on the expression profile of super-enhancer related lncRNAs and mRNAs in cervical cancer HeLa cells
Source: PeerJ. 2024 Feb 23;12:e17035. doi: 10.7717/peerj.17035 (PMC10896078; doi:10.7717/peerj.17035)
Supplement: Supplemental Information 10 [file peerj-12-17035-s010.doc]

# MIAME Checklist

Experiment Design:

1. Goal: To generate the expression profile of cervical cancer (CC) HeLa-cells treated with different concentrations of bromine domain protein 4 (BRD4) inhibitor (JQ1) using whole transcriptome resequencing.
2. Brief description: The potential antitumor effects of JQ1 on cervical cancer cells and its impact on the expression profile remain unknown. To explore the mechanism of JQ1 affecting the progression of cervical cancer, this study used high-throughput sequencing technology to investigate the transcriptome changes of cervical cancer cells treated with JQ1 to reveal the signal pathway and biological function of JQ1 on cervical cancer cells and to provide transcriptome evidence for the application of cervical in the potential treatment of cervical cancer.
3. Keywords: BRD4 inhibitor; cervical cancer; JQ1; HeLa cells; RNA-Seq
4. Experimental factors: 1 μmol/L concentration of JQ1 treated for 72h was applied to inhibit HeLa cells in experimental group, and while the control group was incubated with a drug-free medium for 72 h.
5. Experimental design:

|  | Sample | Biological replicate | Group | Condition | Line |
| --- | --- | --- | --- | --- | --- |
| 1 | 1 | CTL_1 | Control | DMSO | HeLa-cells |
| 2 | 2 | CTL_2 | Control | DMSO | HeLa-cells |
| 3 | 3 | CTL_3 | Control | DMSO | HeLa-cells |
| 4 | 4 | JQ_1_1 | JQ_1 | 1 μmol/L concentration of JQ1 | HeLa-cells |
| 5 | 5 | JQ_1_2 | JQ_1 | 1 μmol/L concentration of JQ1 | HeLa-cells |
| 6 | 6 | JQ_1_3 | JQ_1 | 1 μmol/L concentration of JQ1 | HeLa-cells |

1. Quality control steps taken:

Using fastp software to filter Raw Data, quality control mainly includes the following steps: removing sequencing primers and connectors; removing low-quality sequences at the end; removing sequences with fragment length<50bp.

1. Links to supplemental websites/database accession numbers: this data will be available through GEO at: <http://www.ncbi.nlm.nih.gov/geo/> after applying to upload the raw data.

Samples used, extract preparation and labeling:

1. Origin and characteristics of each sample:

| **Cell Line** | **Tumor Type** |
| --- | --- |
| HeLa-cells | Cervical cancer |

1. Manipulation of biological samples and protocols used: Total cellular RNA was extracted according to the kit instructions, and the RNA purity and concentration were determined using a spectrophotometer and the Qubit RNA assay kit in the Invitrogen Qubit 3.0. Agarose gel electrophoresis (NanoDrop 2000) and Agilent 2100 Bioanalyzer were used to assess the RNA integrity.
2. Experimental factor value for each experimental factor: High-throughput sequencing was performed for cell line sample.
3. Technical protocols for preparing the hybridization: Cell line culture, cDNA Library Construction and Sequencing was performed by standard methods.
4. External controls: Total RNA was quality ascertained using the Agilent Bioanalyser 2100 using the NanoChip protocol. RSeQC software was used to evaluate the quality of transcriptome data, and only the sequencing data qualified in the quality evaluation can reflect the real experimental results in the final results of bioinformatics analysis.

Hybridization Conditions and Parameters:

The protocol and conditions used for hybridization, blocking and washing, including any post-processing steps such as staining: cDNA preprocessing and array hybridization were performed according to the manufacturer’s directions. The detailed protocol is available at the following url: [Technical Bulletins (illumina.com)](https://support.illumina.com/bulletins.html).

Measurement Data and Specifications:

- 1. Raw data: will be available at: <http://www.ncbi.nlm.nih.gov/geo/>) after applying to upload the raw data.
  2. Normalized and summarized data: RNA sequencing libraries were constructed using the TruSeq Stranded Total RNA Library Prep Kit. The constructed libraries were sequenced using the Illumina Novaseq 6000 sequencing platform. The data were collected and processed after sequencing was completed.

Array Design: High-throughput sequencing was completed through the Illumina novaseq 6000 platform.
